# Developments in Taste-Masking Techniques for Traditional Chinese Medicines

**DOI:** 10.3390/pharmaceutics10030157

**Published:** 2018-09-12

**Authors:** Xiao Zheng, Fei Wu, Yanlong Hong, Lan Shen, Xiao Lin, Yi Feng

**Affiliations:** 1 College of Chinese Materia Medica, Shanghai University of Traditional Chinese Medicine, Shanghai 201203, China; 13395830229@163.com (X.Z.); shutcmsl@163.com (L.S.); 2 Engineering Research Center of Modern Preparation Technology of TCM of Ministry of Education, Shanghai University of Traditional Chinese Medicine, Shanghai 201203, China; hfuir@163.com (Y.H.); shutcmfyi@163.com (Y.F.)

**Keywords:** taste-masking techniques, bitterness, compliance, traditional Chinese medicine

## Abstract

A variety of pharmacologically active substances, including chemotherapeutic drugs and the substances from traditional Chinese medicine (TCM), always exhibit potent bioactivities after oral administration. However, their unpleasant taste (such as bitterness) and/or odor always decrease patient compliance and thus compromise their curative efficacies in clinical application. Therefore, the developments of taste-masking techniques are of great significance in improving their organoleptic properties. However, though a variety of taste-masking techniques have been successfully used to mask the unpalatable taste of chemotherapeutic drugs, their suitability for TCM substances is relatively limited. This is mainly due to the fact that the bitter ingredients existing in multicomponent TCM systems (i.e., effective fractions, single Chinese herbs, and compound preparations) are always unclear, and thus, there is lack of tailor-made taste-masking techniques to be utilized to conceal their unpleasant taste. The relevant studies are also relatively limited. As a whole, three types of taste-masking techniques are generally applied to TCM, including (i) functional masking via sweeteners, bitter blockers, and taste modifiers; (ii) physical masking via polymer film-coating or lipid barrier systems; and (iii) biochemical masking via intermolecular interaction, β-cyclodextrin inclusion, or ion-exchange resins. This review fully summarizes the results reported in this field with the purpose of providing an informative reference for relevant readers.

## 1. Introduction

A variety of pharmacologically active substances orally administrated are proved to be quite effective in treating diseases. However, their undesirable organoleptic sensations (e.g., aversive taste and/or odor) always severely compromise their treatment efficiency, especially in pediatrics, owing to the low patient acceptability. Therefore, the developments of taste-masking techniques are of great importance in improving the patient compliance and thus their curative efficacies in clinic.

In recent years, a diverse range of taste-masking techniques have been widely utilized to mask the unpleasant taste of chemical drugs at three levels in the clinic. First, in the formulation level, diverse sweeteners, flavors, and bitter blockers were used alone or in combination to mask such drugs’ taste and/or odor. For example, 0.8% aspartame, a high-potency sweetener, could significantly reduce the bitter taste of 25% acetaminophen in solution [1]. The higher taste-masking efficiency could be achieved by the combination of sweeteners and flavors (for example, the mixture of sodium saccharin and some flavors was used to conceal the bitterness of ibuprofen formulated as syrup with pyridoxine HCl) [1]. This may be owing to the fact that different sweeteners and flavors target different types of taste receptors, and thus, more G proteins are activated to transfer more nerve signals to sweetness-perceived areas in the brain [2]. As for bitter blockers, due to possessing sufficiently strong bitter-masking capability, they are often used alone to inhibit the bitterness of chemical drugs (e.g., γ-amino butyric acid could effectively suppress the unpleasant taste of catechin) [3].

Second, in the particle level, polymer film-coating and lipid barrier systems can impart a physical barrier onto the surface of bitter components to prevent the contacting of drugs and taste receptors in the mouth. In the polymer film-coating, pH-responsive polymers are often used. For example, mequindox-loaded mesoporous silica nanoparticles were coated with a kind of pH-sensitive polymer (Fe-4,4′-bipyridine complex) by virtue of metal–organic coordination cross-linking [4], which showed a rapid drug release at pH 1.0, but a delayed release at pH 6.6. Namely, the entrapped drug could not rapidly diffuse from the surrounding polymer for its bitter sensation to be perceived in the oral cavity. Similar results could be also found in paracetamol-entrapped polymers [5]. To further prevent such drugs from releasing in the mouth and to eradicate their bitterness, a double coating layer consisting of saliva-insoluble Eudragit RS30 as the inner layer and water-permeable Eudragit RL30D as the outer layer was used to coat cetirizine HCl [6]. Moreover, to acquire a better coating effect, microencapsulation was applied to enwrap such drugs (e.g., prednisolone microparticles based on Eudragit E PO or E 100 efficiently masked the drug’s taste and completely and rapidly released the drug at pH = 1.2) [7]. One more advantage for microencapsulation is that the coating layer of microencapsulated taste-masking particles could remain intact during being compacted into orally disintegrating tablets [8]. In lipid barrier systems, various lipids (e.g., glyceryl monostearate, waxes) and solid lipid nanoparticles were utilized as the coating layer or drug carriers to mask the unpleasant taste of these drugs with a solvent-free process [6,9,10].

Third, in the molecular level, charged polymers, β-cyclodextrin, and ion-exchange resins have been used to complex drug molecules with the purpose of masking their taste. For example, cationic Eudragit EPO was used to complex ibuprofen through the process of hot-melt extrusion [11]. The achieved bitterness suppression of ibuprofen was owing to the formation of hydrogen bonding between ibuprofen as a hydrogen donor and the cationic functional tertiary amino of Eudragit EPO as a hydrogen bonding acceptor. In other cases, drug molecules (e.g., cetirizine, diltiazem hydrochloride, dextromethorphan hydrobromide, and tramadol) were enwrapped into the inner cavity of cyclodextrin or adsorbed by charged resins to acquire the taste-masking effect [12,13,14,15].

**Figure 1:**
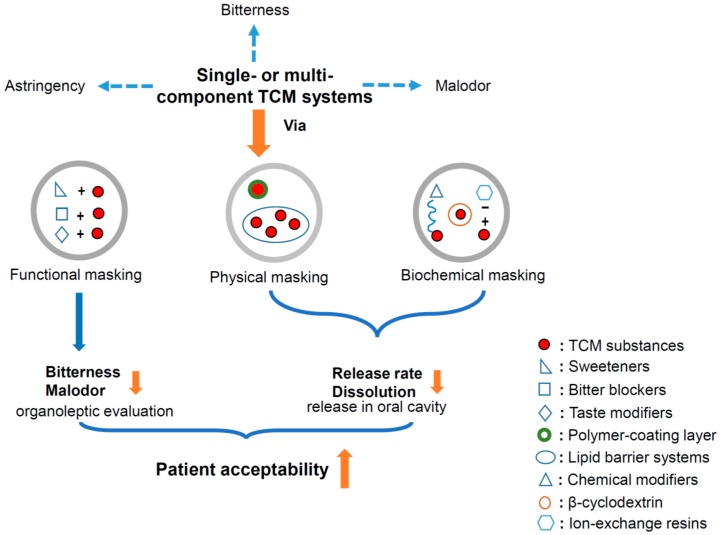
Improvement of patient acceptability of traditional Chinese medicine (TCM) systems via three types of taste-masking techniques.

However, the application of these taste-masking techniques in traditional Chinese medicine (TCM) is relatively premature, though the taste and/or odor of most TCMs are always unpleasant or even repulsive. The types of TCM include bioactive single ingredients, effective fractions, single Chinese herbs, and compound preparations. Excepting the single ingredients, the remaining TCM sources are all multicomponent complicated systems, in which both the bitter components and the structure–activity relationship are generally unclear. Thus, it is very difficult to discover a kind of tailor-made taste-masking technique to cover the unpleasant taste of such systems, and there are also relatively limited studies paying attention to this field. It is so urgent and necessary to do so to improve the patient compliance to and, thus, the unique curative efficacies of TCM in the clinic. In this review, the up-to-date reports in this field were fully summarized with the purpose of providing an informative reference for boosting relevant studies. In general, three types of taste-masking techniques were applied to TCM, including: (i) functional masking via sweeteners, bitter blockers, and taste modifiers; (ii) physical masking via polymer film-coating and lipid barrier systems; and (iii) biochemical masking via intermolecular interaction, cyclodextrin inclusion, and ion-exchange resins (Figure 1) [16,17].

## 2. Functional Masking

**Figure 2:**
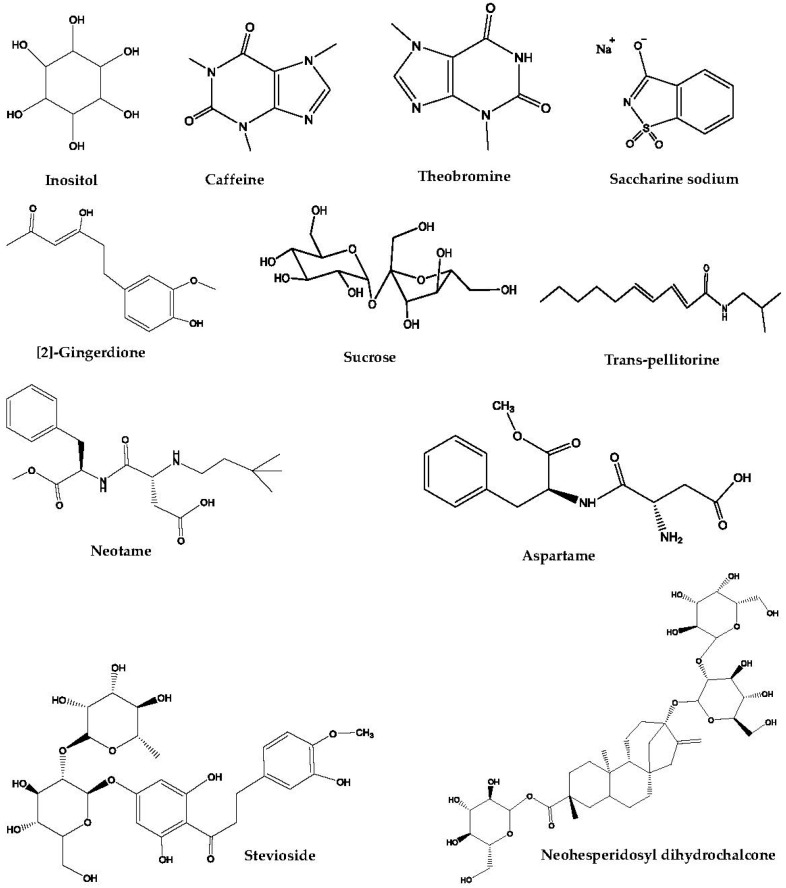
The chemical structures of taste-masking agents involved in functional masking.

Functional masking is the simplest strategy applied in taste masking. Among the agents used, sweeteners, bitter blockers, and taste modifiers are the three important representatives (Table 1, Figure 2). They can be used alone or in combination for the various types of TCM mentioned above.

### 2.1. Sweeteners

Sweeteners are composed of several categories of compounds with different molecular structures, including sugars and polyols (e.g., fructose, sucralose, and mannitol), peptides (e.g., aspartame), and others (e.g., ammonium glycyrrhizate) [18].

Single sweeteners or paired sweeteners can be used to mask the undesirable taste of some bioactive ingredients derived from TCM (e.g., quinine and aristolochic acid). Quinine, one of the bioactive components extracted from the *Cinchonae* cortex, is often used to treat severe malaria (especially for infants) [27]. However, due to its intrinsic strong bitterness, the patient compliance was extremely low in the clinic, resulting in a compromised efficacy of this compound. To suppress the bitterness of quinine, high-potency sweeteners (such as aspartame and saccharine sodium) have been added to the formulation [18]. The sensory tests showed that the bitterness score of quinine hydrochloride (0.1 mM) was significantly decreased after the addition of each of the two sweeteners. Furthermore, the changes in membrane potential caused by the adsorption of both bitterness and sweetness sensors would not be obviously affected by the addition of the sweeteners or the bitter substance (e.g., quinine hydrochloride). Namely, the mechanism of bitter masking of such sweeteners may be due to the fact that the sweet substances activated sweet-taste receptors to produce sweetness so that the sensitivity of taste buds to bitterness was remarkably declined.

In another investigation, three different concentrations of aspartame (0.5, 1, and 2 mmol/L) were added to the quinine solution (15 mmol/L), respectively [19]. The principal component analysis (PCA) results measured by an electronic tongue showed that each of these samples was located in an individual logical order (I, II, III), indicating that the perceived bitterness of quinine could be further decreased with the increasing of aspartame levels. Namely, the added quantities of sweeteners could also have a significant effect on the efficiency of inhibiting the perceived bitterness of quinine. Furthermore, the taste-masking efficiency could be further boosted by virtue of the paired sweeteners. For example, the combination of sucrose and inositol was mixed with aristolochic acid (extracted from *Caulis aristolochiae manshuriensis*). The biting assay of *Manduca sexta* caterpillars showed that the number of bites for the sucrose plus inositol group was the largest, followed by the single sucrose and inositol groups (~17, 11 and 10, respectively). Namely, the paired sweeteners exerted a more potent capability of masking the aversive taste of aristolochic acid [20].

Some sweeteners were also used to mask the aversive taste of several decoctions of TCM as well as TCM compound preparations. For example, two sweeteners (stevioside and neohesperidosyl dihydrochalcone) were added to the water decoction of *Sophorae flavescentis* Radix, respectively [21]. As a result, both the bitter value and bitter level of the decoction containing stevioside decreased. However, as the stevioside level exceeded 0.675%, the bitter value of the decoction no longer descended and even began to ascend. The possible reason may be owing to the fact that the bitterness of stevioside itself will be gradually enhanced as its content increases to a certain level. Namely, the dosage of some sweeteners should be controlled in an appropriate range, or their bitterness-inhibiting efficiency would be reduced. A similar trend was also found in the neohesperidosyl dihydrochalcone group. Moreover, neotame was another sweetener used in some TCM decoctions, including *Scutellariae* Radix, *Coptidis* Rhizoma, *Phellodendri chinensis* Cortex, *Gentianae* Radix et Rhizoma, and *Andrographis* Herba [22]. The assay showed that the bitterness values of these decoctions were all dramatically reduced. In another study, some sweeteners (e.g., xylitol and sucralose) combined with β-cyclodextrin were applied to mask the undesirable taste of compound TCM preparations, such as Xiao’er Xiaoji Zhike Oral Liquid [28].

### 2.2. Bitter Blockers

Bitter blockers are another type of functional masking agent, which can effectively prevent bitter-tasting substances from binding to bitter receptors by means of competitively binding and then blocking the transfer of bitterness signals to the brain.

Several bitter blockers have been used to mask the bitterness of some bioactive TCM components and single Chinese herbs. For example, bitter melon (*Mormordica charantia* L.) is one of the functional vegetables in Asia and also considered as the homology of medicine and food. It has many bioactivities, such as antitumor, anti-inflammation, and antioxidation [29,30]. However, its extreme bitterness, mainly caused by two innate compounds (i.e., momordicosides K and L), made it unpopular in many countries. To tackle this problem, several commercial bitter blockers (such as MR15, 234A, MZ70, and 6100) were added into bitter melon, and then a HPLC-MS method was used to measure the contents of momordicosides K and L [23]. As a result, the samples of bitter melon treated with such blockers showed similar extracted ion chromatograms compared to the untreated bitter melon; that is, the bitterness-masking mechanism of such blockers should be owing to their competitive binding for bitter receptor sites rather than the removal of the two momordicosides.

Intriguingly, some bioactive ingredients themselves can be used as bitter blockers as well. For example, some short-chain gingerdione derivatives, e.g., 1-(4-hydroxy-3-methoxyphenyl)hexa-3,5-dione ([2]-gingerdione), 1-(4-hydroxy-3-methoxyphenyl)hepta-3,5-dione ([3]-gingerdione), and 1-(3-hydroxy-4-methoxyphenyl)hexa-3,5-dione ([2]-isogingerdione), were mixed with quinine [24]. The bitter sensation of quinine (5 mg/kg) containing [2]-gingerdione or [3]-gingerdione was significantly reduced. Additionally, the sucrose equivalents (SEs) of sucrose solutions (5%) containing [2]-isogingerdione were remarkably larger than those obtained by the sum of the SEs of the sucrose solution and [2]-isogingerdione; that is, such gingerdione derivatives could not only mask the bitter sensation of quinine, but also induce the amplification of the sweetness response of sucrose.

### 2.3. Taste Modifiers

Some other taste modifiers can also be applied to the taste-masking of TCM-derived substances by virtue of their effects on taste modification. For example, epigallocatechin-3-gallate (EGCG), a kind of catechin extracted from green tea, was found to possess many health benefits to our bodies (e.g., anti-inflammation and antivirus) [31,32]. However, the taste (i.e., astringency and coexisting bitterness) of EGCG is always perceived as aversive and thus leads to a limited ingestion, especially at higher concentrations [25]. Luckily, several taste modifiers have been found to successfully mask such off-tastes of EGCG [25]. As trans-pellitorine (5 mg/kg) was added to EGCG (750 mg/kg), the astringent intensity of EGCG was obviously reduced along with slightly decreased bitterness as time passed. Namely, the aversive astringency and bitterness of EGCG were both improved, which might be owing to the effect of trans-pellitorine on activating trigeminal neurons. In addition, a similar trend was also found for other structurally related pellitorine derivatives (e.g., cis-pellitorine and (2*S*)-2-[[(2*E*,4*E*)-deca-2,4-dienoyl]-amino]propanoic acid).

In addition, it is well-known that ginseng can effectively benefit our bodies due to the fact that the innate bioactive ginsenosides have the efficacies of inhibiting tumor growth [33] and ameliorating vascular function [34]. However, the peculiar taste of ginsenosides restricted their application in food products [35]. Hopefully, the bitter substances existing in chocolate and coffee might be utilized to mask the off-taste of ginseng. To verify this, Chung et al. added to ginseng extracts three kinds of solutions composed of only caffeine, only theobromine, and the combination of the two components and cyclo (L-Pro–L-Val) (10:6:9 mass ratio), respectively [26]. As a consequence, the decline of the sweetness of ginseng extracts containing the combined substances was the largest in comparison to the two single components (~68.8% vs. 54.2%/43.7%). Therefore, such combined substances may effectively modify the unique taste of ginseng due to the introduction of bitterness to balance the undesirable taste included.

### 2.4. Others

Due to the composition complexity of compound TCM preparations, single taste-masking agents often cannot effectively mask their undesirable tastes. The combination of such agents and flavors appears to be more useful in these cases.

For example, the combination of sucralose and orange flavor (0.15–0.05% ratio of mass fractions) could significantly improve the efficiency of taste-masking of Compound Banlangen Oral Liquid compared to the original recipe (45% sucrose) by means of manual evaluation [36]. In other cases, taste-modifying formulations, alone or in combination with a β-cyclodextrin inclusion technique, could be utilized to further boost the extent and efficiency of taste-masking of some compound preparations (e.g., Kang’erling Granules) [37,38].

## 3. Physical Masking

In the process of physical masking, polymer film-coating and lipid barrier systems can impart a physical obstacle around the TCM-derived bitter substances to avoid them from contacting with taste receptors in the tongue (Table 2).

### 3.1. Polymer Film-Coating

In recent years, some water-insoluble polymers (e.g., acrylate copolymers) and biopolymers (such as chitosan) have been applied to coat some multiple-unit dosage forms (e.g., granules and pellets) of TCM-derived substances (e.g., artemether, dihydroartemisinin, and extracts of *Coptis* Chinensis) and/or microencapsulate bioactive TCM components (e.g., berberine and artemether) for taste-masking via minimizing their release in the oral cavity.

#### 3.1.1. Opadry^®^ Enteric

Some polymer-based materials (e.g., Opadry^®^ Enteric) have been utilized to enwrap the surface of TCM ingredients’ granules (e.g., dihydroartemisinin and artemether) to delay the drug release in the oral cavity with the purpose of taste-masking.

In comparison to conventional tablets, granules, one type of oral multiparticulate forms, have the advantages of (i) being easy for children to swallow; (ii) being able to achieve the aim of individual administration with significantly declined risk of dose dumping; and (iii) being able to be dispersed in the taste-masking suspension matrix to improve the drug mouthfeel [10]. Due to these unique merits, a variety of TCM substances have been prepared into granules.

Dihydroartemisinin (DHA), a derivative of artemisinin isolated from *Artemisia annua*, exhibits a potent antimalarial efficacy with low recurrence and is often prepared into granules [54]. However, the bitter taste of DHA always results in low patient compliance, especially for pediatric patients, and thus reduces its curative efficacy in the clinic [39]. To cover its bitter taste, two polymer-based materials (i.e., Methocil™ E5 and Opadry^®^ Enteric) were used to coat DHA-containing granules [39]. The results of scanning electron microscopy showed that the shape of Opadry^®^ Enteric-coated granules was more regular than that coated with Methocil™ E5. The in vitro dissolution assays illustrated that the DHA-releasing percentage of Opadry^®^ Enteric-coated granules was the lowest (34% ± 3%) in the first 2 min in phosphate buffer (pH 6.8), merely accounting for about 60% and 50% of those obtained from the Methocil™ E5-coated granules and the plain granules, respectively. Namely, a proper coating could protect DHA from escaping early from the granules. Correspondingly, manual taste perception evaluation indicated that the bitter taste intensity score of Opadry^®^ Enteric-coated granules was much lower than that of Methocil™ E5-coated granules (47.3 vs. 80.5), further confirming the superior taste-masking effect of the Opadry^®^ Enteric coating. In another investigation, the same two coating materials were also used to enclose particles of a dry suspension containing another artemisinin derivative (named artemether) for taste-masking [40]. Similarly, the superior taste-masking effect was achieved by the Opadry^®^ Enteric coating. Besides, the stability of artemether was ensured in the reconstituted suspension for seven days (the whole dosing period) and its taste and color were both improved.

#### 3.1.2. Acrylate Copolymers

Some types of acrylate copolymers could be utilized to either coat granules and pellets (multiparticulate dosage forms) of TCM substances or to microencapsulate TCM bioactive components to mask their aversive bitterness.

Acrylic resin II, a type of acrylate copolymers, was found to efficiently coat the granules of some TCM multicomponent systems for taste-masking. For instance, such a polymer was applied to coat the granules of ethanoic extracts of *Coptis* Chinensis [41], leading to a substantial drop in the bitterness of *Coptis* Chinensis along with an improved moisture-proof effect. However, such coated TCM granules could show the following defects: First, the low solubility of the taste-masking granules would easily produce sediment during the administration, leading to a nonuniform dispersion in water and, subsequently, an inaccurate dose. Second, the gritty taste of the remaining sediment would have a significant effect on mouthfeel. To tackle these defects, these TCM granules are often further prepared into suspension particles [42]. In general, for most multicomponent TCM systems, the bitter component included is always indefinite. Therefore, the object of coating should be the whole system, rather than one or more ingredients contained in the system.

Another type of acrylate copolymers (i.e., Eudragit^®^ E PO) was utilized to enclose quinine-containing pellets (another multiparticulate dosage form) [43]. The in vitro dissolution assays showed that the released amount of quinine loaded in coated pellets was markedly reduced in water compared to the uncoated pellets. This result was highly correlated with the bitterness score of the formulations measured through an electronic tongue. Specifically, the bitterness of 10% polymer-coated pellets was acceptable and that of 20%/30% polymer-coated pellets was considered as negligible in comparison to the unacceptable bitterness of uncoated pellets (9.8 vs. <4.5 vs. ≥16.5, intensity score of bitterness). In contrast, in an acid medium, a similar release profile of quinine was achieved by both coated and uncoated pellets. Therefore, such a coating material only created an obstacle between the formulation and the neutral medium to delay the releasing of quinine in saliva (pH 6.8–7.4) [55], but would not impact its release in the gastrointestinal tract. The pharmacokinetic tests indicated that the values of AUC_0–24h_ and C_max_ of quinine achieved by such pellets were both higher than those from the commercially available tablets [44], which were all in the range of quinine previously reported [56,57]. Namely, the coated pellets not only boost the drug bioavailability compared to the tablets, but also could be safe to be applied to children.

Apart from the coating of some TCM-based multiparticulate dosage forms, acrylate copolymers could also be applied to microencapsulate some TCM-derived ingredients for taste-masking by virtue of coating bitter drugs with polymers or filling them into polymeric matrices, thus creating a physical barrier around such drugs to protect them from directly contacting with the taste buds [58,59].

Eudragit E100 (EE), a cationic copolymer, is only soluble in an acid medium (pH < 5) [47,60]. This makes it intact in the oral cavity (with the pH being 5.8–7.4), but quickly dissolved in the stomach (pH 1–3) [45]. Thus, EE could be used to coat TCM substances to mask their undesirable taste by the process of microencapsulation. For example, Shah et al. designed a kind of EE-coated artemether microparticles by the coacervation phase separation method [45]. As expected, the percentage of released drug from the microparticles was significantly less than from the pure drugs at pH 6.8. Moreover, as the amount of EE increased, drug release was further retarded owing to a thicker film formed around the drug particles [61]. Correspondingly, the bitterness score of artemether also declined after the amount of EE increased. However, at pH 1.2, the release of artemether was completed in several minutes, owing to the quick dissolution of EE at acidic pH [62]. Berberine, another bioactive TCM ingredient, has the function of curing acute diarrhea and vasodilation [47,63]. To cover its bitter sensation, berberine microparticles were further prepared into EE-coated microcapsules [46]. The dissolution assays indicated that the percentage of drug release was much higher in hydrochloric acid solution (0.1 mol/L) than in water. Namely, the microencapsulation technique could not only fully mask the bitterness of berberine hydrochloride at oral cavity, but also not influence its dissolution process in the gastrointestinal tract. In another investigation, the taste-covered berberine microcapsules were further formulated into orally disintegrating tablets (ODTs), which could rapidly disintegrate in a few seconds in the oral cavity and then be swallowed directly without the process of chewing or watering, hence being particularly suitable for the patients with swallowing problems (e.g., children and elderly) [47]. The drug release rate from such ODTs in an acid medium was evidently faster than from commercial tablets within the initial 10 min. This should be attributed to both the quick disintegration of drug-loaded ODTs and fast dissolution of the EE coating in an acid medium. In another study, ethyl cellulose was also successfully used to coat total glycosides of *Gentianae* Radix for masking their bitter taste [64].

#### 3.1.3. Biopolymers

Some biopolymers (e.g., chitosan and alginate), due to their excellent features (e.g., biodegradability, biocompatibility, and low toxicity), are found to be suited to microencapsulate the TCM-derived substances with the purpose of masking their unpalatable tastes [65,66].

For example, Shah et al. prepared an artemether-entrapped chitosan microparticle using sodium hydroxide (NaOH) as the crosslinking agent [48]. By the formulation optimization, the particle size, encapsulation efficiency, and drug release of the microparticles all achieved the desired characteristics. As a whole, the percent of artemether release was much lower than that obtained by pure artemether at pH 6.8. Moreover, as the quantity of the crosslinking agent increased, the drug release further decreased. With the increase of crosslinking, the swelling capability of chitosan was essentially reduced, which could effectively protect the physical barrier around the drug from being degraded and thus improve the efficiency of taste-masking. Specifically, the bitterness score of artemether was reduced to 0, compared to over 3 for the pure drug. Namely, such chitosan-based microparticles fully masked the bitterness of encapsulated artemether. A similar result was also found in the taste-masking of mefloquine hydrochloride [67].

In addition, to further prevent the bioactive constituents from probable chemical degradation, chitosan was combined with another biopolymer, alginate, to microencapsulate the oleaginous fraction obtained from the fruit of *Pterodon pubescens* Benth [49], which has a variety of bioactivities, such as antinociception [68], antileishmaniasis [69], and antiproliferation [70]. By virtue of measuring the concentration of four bioactive components (vouacapan 1–4) included in the fraction, the microcapsules comprised of alginate and low-molecular-weight chitosan showed the largest microencapsulation efficiency, compared to those consisting of alginate and medium-molecular-weight chitosan or alginate and chitosan with more than 75% deacetylation (61.01 ± 2.00% vs. 47.07 ± 2.01% or 57.17 ± 1.63%). Moreover, the residual quantity percentage of the microencapsulated oleaginous fraction was significantly higher than that of the free fraction as the temperature was increased to 500 °C, suggesting that the process of microencapsulation could effectively prevent such an oleaginous fraction from degradation. More importantly, the microencapsulated oleaginous fraction could release rapidly in stomach acid, but was slowly released in the oral cavity, hence effectively concealing its undesirable taste without the marked sacrifice of its oral absorption. In general, the swelling extent of the alginate/chitosan complex is highly dependent on the pH value. At pH < 2, fast release of the oleaginous fraction resulted from the marked swelling of the alginate/chitosan complex, due to the protonation of chitosan. At pH > 5, the slow release resulted from the slight swelling of the complex, owing to the ionized carboxylic group of alginate [71]. In another investigation, polymerized whey protein was also successfully used to microencapsulate ginsenosides with the purpose of taste-masking [72].

### 3.2. Lipid Barrier Systems

Lipid barrier systems can also be applied to encapsulate the TCM-derived substances for taste-masking. Compared with the polymer-film coating, they exhibit many advantages, including: (i) simplifying the coating process by virtue of only melting the lipid excipients prior to applying (hence, no solvent is required); (ii) reducing the usage of both the type and amount of excipients; (iii) protecting the coating film from cracking during compaction due to its plastic nature; (iv) reducing the cost of production due to the relatively lower price of lipid excipients [10].

Hot-melt extrusion is the common technique used to enclose pharmacologically active ingredients (API) within lipid excipients. In this process, API could be mixed with melting lipid excipients during the melt stage and then be extruded to form a certain dosage form (e.g., granules). After cooling and solidification, API could be coated by the shell of lipid excipients or entrapped within the matrix [50,73].

Recently, by virtue of this technique, some lipid barrier systems have successfully enclosed TCM-derived bioactive ingredients (e.g., quercetin and curcumin) to cover their undesirable taste and improve their dissolution profile. For example, quercetin, a flavonoid extracted from several TCMs (such as *Sophorae* Flos), has excellent bioactivities, such as anti-inflammation, antioxidation, and antiobesity [74,75]. However, its poor dissolution behavior and bitter taste both restrict its clinical application. To overcome these, Khor et al. used carnauba wax to microencapsulate quercetin powders via the hot-melt extrusion technique [50]. As a result, in the medium of pH 6.8, the dissolution percentage of quercetin was essentially reduced. This should be due to the hydrophobic nature of the wax [76]. Correspondingly, the bitterness value of quercetin was also significantly reduced; that is, the lower amount of quercetin released from the microencapsulated powers made the tongue unable to fully perceive the bitterness of the drug. By contrast, in simulated gastrointestinal fluids, the microencapsulated powders exhibited a comparable dissolution profile with the free drug; in other words, the microencapsulated powders not only concealed the bitterness sensation of quercetin in the oral cavity, but also did not influence the dissolution profiles of the drug in the gastrointestinal tract, and thus preserved its therapeutic efficacy.

In another study, to achieve taste-masking and enhanced bioavailability, Chuah et al. prepared an amorphous solid dispersion of curcumin entrapped in a matrix (composed of lecithin, isomalt, and hydroxypropyl methylcellulose), employing the hot-melt extrusion technique [51]. The in vivo pharmacokinetics (PK) studies suggested that the AUC_0–∞_ and C_max_ values of the solid dispersion were 13-fold and 7-fold larger than those obtained by the free drug, respectively; that is, the bioavailability of curcumin was significantly improved by such a solid dispersion form. This should be due to the fact that (i) crystalline curcumin was transferred into the amorphous form in the matrix, resulting in an apparent increase in its solubility [77]; and (ii) the application of lecithin improved the drug’s capability of permeating into the intestinal membrane and also enhanced its bioadhesion to the wall of the gastrointestinal tract [51]. At the same time, the amorphous solid dispersion of curcumin also improved the undesirable taste, smell, and color of the free drug.

In addition, solid lipid micro- and nanoparticles are also used for the same purpose. For example, the proanthocyanidin-rich cinnamon extract has many bioactivities (e.g., anti-inflammation, antioxidation, and cardioprotection), especially applicable to prevent the damage induced by diabetes [52,78]. However, the low stability and unpleasant taste of proanthocyanidins strictly limit its application in medical and food products [79]. To tackle this, Tulini et al. developed a kind of solid lipid microparticles (SLMs) to encapsulate the cinnamon extract using the spray chilling technique [52]. The stability assays showed that the content of proanthocyanidins was maintained in a relatively high percentage (over 82%) during 90 days’ storage at different temperatures. More importantly, the human sensory tests indicated that the physical mixture of the extract and the blank SLMs was both bitterer and more astringent than the extract-containing SLMs; that is, the taste and stability of such a cinnamon extract were both improved by means of loading it into SLMs. In another investigation, with the aim of enhancing the dosing accuracy of quinine in pediatrics and masking its bitterness, Dandagi et al. prepared a type of quinine sulphate-loaded solid lipid nanoparticles (SLNs) [53]. The released percentage of quinine from SLNs was much lower than from the pure drug in the simulated salivary fluid. In contrast, in 0.1 N HCl buffer (pH 1.2), the release profiles of the SLNs and the pure drug were similar. Therefore, it was desirable for quinine to be delivered by SLNs in terms of not only masking its bitterness in oral cavity, but also not delaying its oral absorption. Moreover, such SLNs could be administrated accurately to children (pediatric dose: 10 mg/kg) due to its relatively low drug content per dispersion volume (~30 mg/mL).

In summary, diverse means of evaluation of bitter taste (e.g., electronic tongue, manual taste, dissolution studies, and in vivo process) have been used alone or in combination with each other to evaluate the extent of the bitter taste of pharmaceuticals before and after taste-masking. In the three types of taste-masking techniques, various means of evaluation are combined together to be used in physical and/or biochemical masking, whereas only sensory evaluations (i.e., electronic tongue, manual taste) are utilized in functional masking. For example, in physical masking, coated and/or loaded TCM substances show a reduced release in a neutral medium, while showing an increased and complete release profile in an acid medium, and thus, the inherent bitterness could be significantly weakened in the oral cavity. This result is always in accordance with the consequence of the sensory tests (i.e., PCA of electronic tongue and manual taste tests). On the other hand, the release rate in the gastrointestinal tract and the in vivo PK profiles of these TCM substances is almost equal to the untreated ones. 

## 4. Biochemical Masking

Some TCM-derived bioactive components (e.g., EGCG, artemether, and andrographolide) could be conjugated by some biological macromolecules (e.g., β-casein and β-lactoglobulin), adsorbed by ion-exchange resins, or enwrapped by β-cyclodextrin to form either poorly water-soluble complexes or drug-loaded inclusions to mask their unpleasant taste (Table 3).

### 4.1. Intermolecular Interaction

Some biological macromolecules can specifically bind with several TCM-derived bioactive components by virtue of forming intermolecular covalent bonds or hydrogen bonding to produce certain poorly water-soluble conjugates.

So far, it has been found that several proteins (such as β-lactoglobulin and β-casein) could be covalently linked with some TCM-derived ingredients (e.g., EGCG, allicin, and quinine) with the purpose of modifying their unpleasant taste or odor. For example, to reduce the bitterness sensation of EGCG and improve its consumer acceptability, Bohin et al. prepared several types of complexes comprised of a diverse range of proteins covalently bound to EGCG [80]. The cell-based receptor assays by an ultrafiltration method showed that the reduction percentage of activation of the bitter receptor hTAS2R39 treated with the complex of β-casein and EGCG was the highest among the four EGCG complexes composed of β-casein, β-lactoglobulin, gelatin B1, and gelatin F1, respectively, (72.8% vs. 5.8% vs. 18.3% vs. 30.6%). Moreover, the binding affinity value of β-casein for EGCG was larger than those obtained by gelatin B1 and β-lactoglobulin. Namely, the tight binding of β-casein to EGCG could effectively inhibit the activation of bitter receptors induced by EGCG and thus remarkably cover the bitterness sensation of EGCG. In addition, the human sensory analysis indicated that as the content of Na-caseinate (a replacer of pure β-casein in food products) was raised to 0.25% (*w*/*v*), the bitterness score of EGCG was significantly decreased from about 7 to 4 due to binding to the protein.

In another study, with the aim of masking the malodorous odor and taste of allicin (a major thiosulfinate from fresh crushed garlic) and enhancing its stability [88,89], Wilde et al. used the β-lactoglobulin (β-LG) to covalently bind with allicin [81]. As a result, after allicin was modified with β-LG, the abundance of diallyl disulfide (DADS) was significantly decreased after spray drying compared to the drying of the unbound mixture of β-LG and allicin (~100 vs. ~13,400), assessed by headspace GC–MS, which proved the successful binding between allicin and β-LG. Besides, the β-LG–allicin complex also exhibited good stability during the drying process. The assays of sensory evaluation showed that as allicin was covalently modified with β-LG to form S-allylmercaptocysteine (SAMC), the taste intensity of fresh garlic aqueous solutions significantly declined. In general, the produced SAMC was buried inside the β-LG, and thus was much less reactive than the original allicin in activating the transient receptor potential family of cation channels to mediate pungency [89]. The further mechanism may be that the SAMC was chemically bound into the polypeptide chain of β-LG, and thus remarkably reduced its volatility compared to the original allicin [90]. Additionally, some researchers also successfully used β-LG to covalently bind with quinine with the purpose of masking its bitter taste [91].

Long-chain fatty acids (≥12 carbon atoms) have also been utilized to interact directly with some TCM-derived ingredients to mask their bitter taste. In order to clarify the mode of interaction and the bitterness-masking mechanism, Ogi et al. initially used isothermal titration calorimetry (ITC) to screen out the bitter components interacting with sodium oleate, and then determined the mode of interactions by nuclear magnetic resonance (NMR) [82]. The ITC results showed that as the selected quinine hydrochloride was titrated with diverse fatty acids (e.g., lauric acid, oleic acid, and linoleic acid), the solutions slowly became turbid. Namely, insoluble quinine–fatty acid complexes were formed in this process. Furthermore, the ^1^H NMR assays revealed that the complexation was attributed to the formation of intermolecular hydrogen bonding between carboxyl groups of fatty acids and nitrogen atoms of quinine. In other words, such binary complexes formed by intermolecular hydrogen bonding could significantly reduce the extent of dissolution of quinine in the oral cavity through the hydrophobic effect, and thus effectively cover the bitter taste of quinine due to the reduced contact between the drug and taste buds.

### 4.2. Cyclodextrin Inclusion

So far, cyclodextrin inclusion techniques have been successfully applied to enwrap some TCM-derived bioactive ingredients as well as multicomponent TCM systems (such as effective fractions), with the aim of masking their unpalatable taste by virtue of temporarily locking or caging them within the internal cavity of cyclodextrin to impede their interaction with the taste receptors on the tongue [83,84].

β-cyclodextrin (β-CD) is the most commonly used cyclodextrin for this purpose. It has been used alone or in combination with another polymer to encapsulate several TCM ingredients (e.g., artemether and thymol) to conceal their bitter taste and boost their dissolution rate. For example, Shah et al. developed a kind of inclusion complex of β-CD and artemether [83]. The apparent solubility of artemether was increased by about 3-fold as the concentration of β-CD was increased from 2 mM to 14 mM. Furthermore, the in vitro release assays suggested that the released percentage of the complex at pH 1.2 and 6.8 were both significantly higher than those of the free drug, which also revealed that the released percentage of the inclusion complex at pH 6.8 was only about 30% of that at pH 1.2. Namely, the inclusion complex not only effectively enhanced the dissolution of artemether in aqueous solutions, but also showed the potential to mask its bitter taste in the oral cavity. This was subsequently confirmed by the tests of gustatory sensation, which illustrated that tasteless bitterness was experienced by all 20 trained volunteers in the complex group, while strong bitterness and very strong bitterness were perceived by 18 and 2 volunteers, respectively, in the free drug group. In general, the improved dissolution should be owing to the molecularly dispersed drug and the higher solubility of the complex compared to the free drug [92]. Moreover, the reduction in the bitterness of artemether should be attributed to the fact that the hydrated outer surface of the inclusion complex effectively prevented the artemether molecule from binding to the G protein-coupled receptors on the apical membrane of taste cells to induce a bitter sensation. Similar results were also obtained with the β-CD complex of hydrophilic primaquine [93].

In addition, to further improve the efficiency of taste- and odor-masking of volatile thymol extracted from the plant of *Thymus mongolicus*, the β-CD complex of thymol was further mixed with a methacrylate copolymer, Eudragit^®^ E PO [84]. As a result, the volatility of thymol was reduced by 11.5 ± 4.9% via β-CD complexation alone and by 96.6 ± 0.5% via β-CD complexation plus mixing with Eudragit^®^ E PO; namely, dispersion of the inclusion complex in such polymers could substantially conceal the undesirable smell and taste of thymol and thus boost the palatability of this volatile drug. The in vitro release results suggested that in the gastrointestinal tract-simulating fluid, the release rate of the inclusion complex dispersed in the copolymer was much quicker than that of the free drug. Namely, the rates of dissolution and absorption of thymol were both evidently increased by the effect of solubilization of β-CD on thymol and the highly dispersed state of the complex in the polymer [94]. In other words, such β-CD complexation plus dispersion in Eudragit^®^ E PO not only thoroughly covered the undesirable smell and taste of thymol, but also improved its dissolution and absorption profiles. In another study, β-CD was also used in combination with another biopolymer, chitosan, to encapsulate aloe and gentian for fully masking their bitter sensation [95].

Besides being applied to mask the taste of TCM-derived effective ingredients, β-CD inclusion techniques can also be used to enwrap multicomponent TCM systems (e.g., effective parts and compound preparations) for taste-masking. For example, as 3% β-CD was added to the decoction of *Andrographis* Herba or *Nelumbinis* Plumula, the level of bitterness of the decoction scored by volunteers was remarkably decreased [85]. Similar results were also found for the extract of *Gentianae* Radix et Rhizoma [96]. In another investigation, β-CD was utilized to encapsulate a compound TCM preparation, Liu-Shen powder [86]. The results of principal component analysis measured by electronic tongue suggested that the relative distance between the β-CD–Liu-Shen inclusion complex manufactured by the colloid grinding method and the original Liu-Shen powder was much higher than that between the complex obtained by the ball grinding method and the original powder (227 vs. 168). Namely, a higher taste-masking efficiency was achieved by preparing the inclusion complex via the colloid grinding method. Moreover, the inclusion percentages of two bioactive components of *Bufonis* venenum (i.e., cinobufagin and resibufogenin) achieved by the colloid grinding method were both higher than those achieved with the ball grinding method. Namely, the tendency of inclusion was in accordance with that of taste-masking.

### 4.3. Complexation with Ion-Exchange Resins

Ion-exchange resins, a kind of reticular and insoluble inert polymers modified with functional moieties, can not only electrostatically bind with the ionized drugs to cover their unpleasant taste in the oral cavity, but also release them by the ionic exchange process in the gastrointestinal tract to improve the bioavailability of such drugs [87].

So far, ion-exchange resins have been widely applied to conceal the undesirable taste of various TCMs (e.g., quinine, andrographolide, and the total glycosides extract of *Gentianae* Radix). For example, Xiang et al. developed a kind of resin complex consisting of the total glycosides extract of *Gentianae* Radix and anionic Amberlite IRA-400 [87]. The optimized preparation parameters were as follows: solution concentration, 8 mg/mL; ratio of resin to drug, 1.5:1; reaction temperature, 45 °C. The bitter level of the optimized resin complex orally assessed by 20 volunteers was zero, comparable to the empty resin, suggesting that the bitter components in the extract were effectively adsorbed by the resin.

In addition, several different types of cationic exchangers were utilized to bind with quinine [27]. As a result, the quinine level in solution was constantly reduced when Amberlite™ IRP88 was used, while it fluctuated when INDION 234 or Amberlite^TM^ IRP69 was used. Moreover, Amberlite^TM^ IRP88 also showed the shortest time for adsorbing half of the amount of quinine. Namely, among the three exchangers studied, Amberlite^TM^ IRP88 was superior in adsorbing the drug ion and masking its bitterness. Similarly, the unpleasant taste of andrographolide was also successfully masked by the ion-exchange resin [97].

## 5. Conclusions

Three types of taste-masking techniques (i.e., functional masking, physical masking, and biochemical masking) have been applied to conceal the unpleasant taste and/or odor of TCM-derived substances with some success. Among them, biochemical masking is mainly used for bioactive TCM-derived ingredients, while the others are suitable for both single TCM ingredients and multicomponent TCM systems.

Functional masking, as the simplest one, is often used to conceal some undesirable tastes of TCM substances. It has been clearly proven that adding sweeteners, bitter blockers, and/or taste modifiers (three basic types) into TCM formulations could significantly weaken their bitterness sensation. In general, the effect of functional masking should be due to the fact that (i) the sweeteners can activate sweet receptors to produce a sweet taste and thus balance the bitter taste of TCM substances; (ii) the bitter blockers can competitively bind with the bitter receptors and thus reduce the frequency of transduction of bitter signals; and (iii) the taste modifiers can improve some unpleasant tastes of TCM substances by virtue of their taste-modifying effects.

As for physical masking, either a polymer film or a lipid barrier can be used to enwrap TCM-derived substances. Such enwrapped TCM substances generally show markedly reduced drug release in the oral cavity, but full release in the gastrointestinal tract due to rapid dissolution/erosion of the polymer/lipid barrier. Therefore, the in vivo PK profiles of such enwrapped TCM substances are almost equal to those of the untreated ones. Namely, such physical masking not only successfully masks the unpleasant taste of TCM substances in the oral cavity, but also does not affect their subsequent gastrointestinal dissolution and absorption. On the other hand, just like functional masking, physical masking is suitable for multicomponent TCM systems, due to the fact that the bitter components existing in such complicated TCM systems are always unclear and yet extremely difficult to be fully identified. Therefore, enwrapping the multicomponent systems as a whole is more feasible for concealing their tastes.

Compared to the former two, biochemical masking is mostly used for the TCM substances with clear bitter ingredients. Specifically, some biological macromolecules and ion-exchange resins can bind with some TCM ingredients by virtue of either intermolecular interactions or electrostatic adsorption to produce poorly water-soluble complexes and thus mask their bitterness sensation in the oral cavity.

In other words, compared with the diverse range of taste-masking techniques applicable to chemical drugs and single bioactive TCM ingredients, the techniques suitable for multicomponent TCM systems are still quite limited. This has already resulted in the reduced clinical application of such multicomponent systems. With further studies on both the bitter TCM components and the taste-masking technologies, tailor-made taste-masking techniques might be discovered for application to these complicated TCM systems for significantly improving their palatability to a degree acceptable by more patients in the clinic.

## Figures and Tables

**Table 1:** Taste-masking of substances derived from traditional Chinese medicine via functional masking.

Types	Masking Agents	Masked Substances	Improved Properties	References
Adding sweeteners	Aspartame; saccharine sodium	Quinine	The bitterness score ↓ 87%, the perceived bitterness further ↓ as the amount of aspartame ↑; CPA value not changed	[18,19]
	Sucrose plus inositol	Aristolochic acid	The biting number of *Manduca sexta* caterpillars treated with the sucrose plus inositol group ↑	[20]
	Stevioside	Decoction of *Sophorae flavescentis* Radix	The decoctions: bitter value ↓ 0.58–1.26 fold, bitter level from IV to III, bitterness ↓ 14.0–30.5%	[21]
	Neohesperidosyl dihydrochalcone	Decoction of *Sophorae flavescentis* Radix	The decoctions: bitter value ↓ 0.44–1.50 fold, bitter level from IV to III, bitterness ↓ 10.65–36.32%	[21]
	Neotame	Decoction of *Scutellariae* Radix, *Coptidis* Rhizoma, *Phellodendri chinensis* Cortex, *Gentianae* Radix et Rhizoma, and *Andrographis* Herba	The bitterness of such decoctions ↓ 70.11%, ↓ 49.12%, ↓ 71.88%, ↓ 50.87%, ↓ 38.39%, respectively; the bitter value ↓ 1.22-fold, ↓ 1.78-fold, ↓ 1.77-fold, ↓ 2.02-fold, ↓ 1.43-fold, respectively	[22]
Adding bitter blockers	MR15, 234A, MZ70, and 6100	Bitter melon	The content of momordicosides K and L treated with such blockers was not changed	[23]
	Gingerdione derivatives	Quinine	The bitterness of quinine ↓ about 20%; the sucrose equivalents of sucrose ↑ about 0.4–1.6%	[24]
Adding taste modifiers	Trans-pellitorine	EGCG	The absolute astringency of EGCG ↓ 18–33%; the absolute bitterness ↓ 3–23%.	[25]
	Caffeine, theobromine, and cyclo (L-Pro–L-Val)	Ginseng	The sweetness value ↓ ~70%	[26]

Note: CPA, the change in the membrane potential caused by adsorption; EGCG, epigallocatechin-3-gallate.

**Table 2:** Taste-masking of substances derived from traditional Chinese medicine via physical masking.

Types	Coating Material	Masked Substances	Dosage Form	Formulation Composition	Preparation Method	Improved Properties	References
Polymer film-coating	Opadry^®^ enteric	Dihydroartemisinin	Granules	Dihydroartemisinin, carbopol, sodium metabisulphite, methyl paraben sodium (14:1:2:3 mass ratio) plus distilled water and Opadry^®^ enteric	Using conventional coating pans	The Opadry^®^ enteric-coated granules: in vitro dissolution of bitter dihydroartemisinin ↓~50% in pH of 6.8; thermal stability of coated granules close to the pure granules	[39]
	Opadry^®^ enteric	Artemether	Particles of dry suspension	Artemether, carbopol 934P, sodium metabisulphite, methyl paraben sodium (7:5:12:16 mass ratio) plus distilled water, Opadry^®^ enteric, sugar fine, aerosil, xanthan gum, tit. Dioxide, orange flavor, NaCl, and citric acid	Using conventional coating pans	The Opadry^®^ enteric-coated particles of dry suspension: in vitro dissolution of bitter artemether ↓ ~50% in pH of 6.8, the bitter taste intensity score of artemether ↓ ~55%	[40]
	Acrylic resin II	Coptis Chinensis	Granules	Coptis Chinensis:starch, microcrystalline cellulose:lactose, Acrylic resin II, aspartame, PEG6000, talc powder	Using conventional coating pans	The coated granules: bitterness ↓; moisture-proof ability ↑	[41]
	Eudragit E100	Andrographitis compound particle	Suspension particles	Sucrose, 10% starch slurry, Andrographitis compound particle, Eudragit E100	Using fluidized bed systems	The coated suspension particles: mouthfeel ↑; dispersed uniformly in water	[42]
	Eudragit^®^ E PO	Quinine sulfate	Pellets	Eudragit^®^ E PO, quinine sulphate pellets, sodium lauryl sulphate, stearic acid, magnesium stearate	Using fluidized bed systems	The coated pellets: in vitro release in water ↓ ~34–85% than uncoated pellets; in vivo AUC_0–24h_ ↑ 0.16-fold, C_max_ ↑ 0.27-fold compared to commercial tablets	[43,44]
	Eudragit E100	Artemether	Microparticles	Eudragit E100, artemether, sodium hydroxide	Using a coacervation phase separation method	The microparticles: high drug loading; rapid release at pH 1.2, release ↓ ~13% in 1 h at pH 6.8; the bitterness score ↓ at pH 6.8	[45]
	Eudragit E100	Berberine	Microcapsules	Berberine hydrochloride, Eudragit E100	Using fluidized bed systems	The microcapsules: nearly 80% dissolved in 0.1 mol/L HCl in 30 min, whereas only ~1% in water in 1 h	[46]
	Eudragit E100	Berberine	Orally disintegrating tablet	Berberine hydrochloride, Eudragit E100, 6% (*w*/*w*) crospovidone XL and 15% (*w*/*w*) microcrystalline cellulose		The ODTs: faster release of the drug than commercial tablets within the initial 10 min in HCl; at 5 min, the percentage of drug release ↑ 1.5-fold compared to common tablets; bioequivalent to the commercial tablets; stable throughout storage of 6 months	[47]
	Chitosan	Artemether	Microparticles	Artemether, chitosan and sodium hydroxide were 0.056 g, 0.03 g and 15 mL	_	The microparticles: the release at pH 6.8 ↓; the release ↓ as the quantity of crosslinking agent ↑	[48]
	Alginate/chitosan complex	Alcohol extract of the fruit of *Pterodon pubescens* Benth	Microcapsules	2.50% alginate solution 0.25% Tween 80 (*w*/*w*) and 72.13% FHPp 0.10% chitosan solution	Using a complex coacervation method	The microcapsules: high encapsulation efficiency; mass loss of 80.40% compared to 99.89% of the fraction alone at 500 °C; 53.85% released at pH = 1.2, 22.03% released at pH = 6.8	[49]
Lipid barrier systems	Carnauba wax	Quercetin	Powders	70% quercetin, 30% carnauba wax/shellac	Using hot-melt extrusion	The powders: dissolution ↓ 0.8-fold compared to the free drug in the oral cavity; the bitterness sensory output ↓ 80%; similar release profile with the free drug	[50]
	HPMC, lecithin, isomalt	Curcumin	Solid dispersion	Curcumin, HPMC, lecithin, isomalt (2:15:2:1)	Using hot-melt extrusion	The solid dispersion: C_max_ ↑ 5.8-fold, AUC_0–∞_ ↑ 11.8-fold, T_max_ ↑ 2.9-fold compared to the free drug; enhanced anti-inflammatory bioactivity at 10-fold lower dose	[51]
	Vegetable fat	Proanthocyanidin-rich cinnamon extract	Solid lipid microparticles	Proanthocyanidin-rich cinnamon extract, vegetable fat (8:72)	Using spray chilling technique	The solid lipid microparticles: ↑ stability; masked the taste of bitterness and astringency	[52]
	Glyceryl monostearate	Quinine sulphate	Solid lipid nanoparticles	Glyceryl monostearate, drug (3:1) + 2% poloxamer 188	Using ultrasonic solvent emulsification technique	The solid lipid nanoparticles: provided accurate dosage to children; quickly released in pH 1.2; ↓ ~100-fold release in pH 6.8	[53]

Note: AUC, area under the concentration–time curve; C_max_, maximum plasma concentration; ODTs, orally disintegrating tables; HPMC, hydroxypropyl methyl cellulose; T_max_, time to reach C_max_; FHPp: the fruit of *Pterodon pubescens* Ben.

**Table 3:** Taste-masking of substances derived from traditional Chinese medicine via biochemical masking.

Types	Complexing Agents	Masked Substances	Preparation Method	Improved Properties	Reference
Intermolecular interaction	β-casein	EGCG	Using ultrafiltration method	The complexes: ↓ 72.8% of the activation of hTAS2R39; binding affinity ↑, maximal binding capacity ↑; the bitterness score ↓ 3	[80]
	β-lactoglobulin	Allicin	-	The complexes: taste intensity ↓ 3, DADS abundance ↓ 1300	[81]
	Sodium laurate	Quinine	-	The binary insoluble complexes ↓ bitter taste	[82]
β-cyclodextrin inclusion	β-cyclodextrin	Artemether	Using physical mixing method	The inclusion complexes: solubility ↑ 3-fold, the release in pH 6.8 ↑ 0.76-fold, the release in pH 1.2 ↑ 6.79-fold; the bitterness score ↓ 3	[83]
	β-cyclodextrin plus Eudragit^®^ E PO	Thymol	Using sealed-heating method	The complexes: volatility ↓ 96.6%; release rate ↑ 7-fold in gastrointestinal fluid, in vitro dissolution rate ↑; T_max_ ↓ 1.29-fold, in vivo absorption rate ↑	[84]
	β-cyclodextrin	Decoction of *Androgra-phis* Herba and *Nelumbinis* Plumula	Using physical mixing method	The level of bitterness of *Andrographis* Herba ↓ 3.5; the level of bitterness of *Nelumbinis* Plumula ↓ 3	[85]
	β-cyclodextrin	Liu-She powder	Using colloid grinding method	The inclusion complexes: relative distance ↑ 59, the inclusion rates of *Bufonis* Venenum ↑ compared to the ones achieved by the ball grinding method	[86]
Ion-exchange resins complexation	Amberlite IRA-400	*Gentianae* Radix total glycosides extract	Using electrostatic attraction method	The resin complexes ↓ bitterness	[87]
	Amberlite^TM^ IRP88	Quinine	Using electrostatic attraction method	The time of quinine adsorbed by the resin ↓ compared to INDION 234 and Amberlite^TM^ IRP69; the drug amount constantly decreased during binding to this resin	[27]

Note: EGCG, epigallocatechin-3-gallate; DADS, diallyl disulfide.
